# Cytokine-Modulating Strategies and Newer Cytokine Targets for Arthritis Therapy

**DOI:** 10.3390/ijms16010887

**Published:** 2014-12-31

**Authors:** Shivaprasad H. Venkatesha, Steven Dudics, Bodhraj Acharya, Kamal D. Moudgil

**Affiliations:** Department of Microbiology and Immunology, University of Maryland School of Medicine, 685 W. Baltimore Street, HSF-1, Suite 380, Baltimore, MD 21201, USA; E-Mails: hvshivaprasad@gmail.com (S.H.V.); sdudics1@gmail.com (S.D.); bacharya@som.umaryland.edu (B.A.)

**Keywords:** autoimmunity, arthritis, biologics, cytokines, gene therapy, inflammation, interleukins, rheumatoid arthritis, siRNA

## Abstract

Cytokines are the key mediators of inflammation in the course of autoimmune arthritis and other immune-mediated diseases. Uncontrolled production of the pro-inflammatory cytokines such as interferon-γ (IFN-γ), tumor necrosis factor α (TNFα), interleukin-6 (IL-6), and IL-17 can promote autoimmune pathology, whereas anti-inflammatory cytokines including IL-4, IL-10, and IL-27 can help control inflammation and tissue damage. The pro-inflammatory cytokines are the prime targets of the strategies to control rheumatoid arthritis (RA). For example, the neutralization of TNFα, either by engineered anti-cytokine antibodies or by soluble cytokine receptors as decoys, has proven successful in the treatment of RA. The activity of pro-inflammatory cytokines can also be downregulated either by using specific siRNA to inhibit the expression of a particular cytokine or by using small molecule inhibitors of cytokine signaling. Furthermore, the use of anti-inflammatory cytokines or cytokine antagonists delivered via gene therapy has proven to be an effective approach to regulate autoimmunity. Unexpectedly, under certain conditions, TNFα, IFN-γ, and few other cytokines can display anti-inflammatory activities. Increasing awareness of this phenomenon might help develop appropriate regimens to harness or avoid this effect. Furthermore, the relatively newer cytokines such as IL-32, IL-34 and IL-35 are being investigated for their potential role in the pathogenesis and treatment of arthritis.

## 1. Introduction

**Figure 1:**
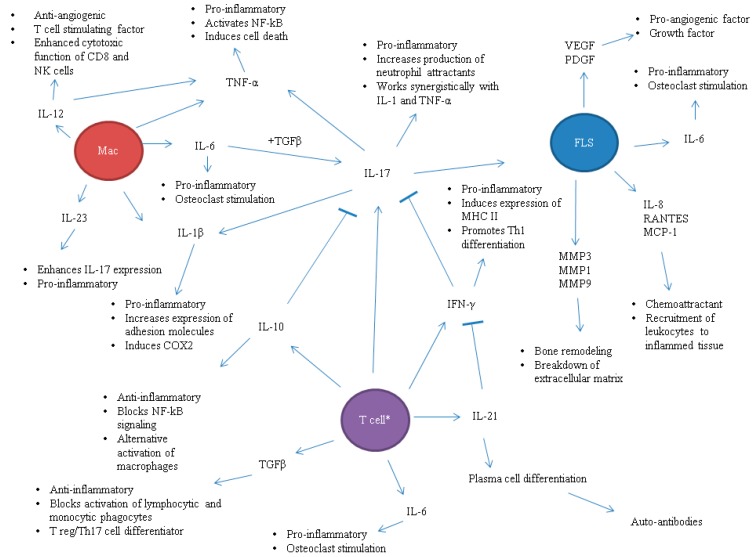
The roles of cytokines in arthritis pathogenesis. The cytokine environment in the joints and the draining lymphoid tissue in rheumatoid arthritis is rather complex. There are multiple cell types that are present and each secretes a panel of pro-/anti-inflammatory cytokines, chemokines, or other inflammatory mediators. Each cytokine has its own role in either promoting an immune response or regulating the immune response. Moreover, one cytokine can have more than one function, known as pleiotropy, or have duality of action (both pro- and anti-inflammatory properties). Furthermore, there is redundancy meaning that there are overlapping characteristics between different cytokines (e.g., IL-6 and IL-17 being pro-inflammatory). Depending on the proportion of cell types present within the joints and the type of immune stimuli that they are exposed to, the overall milieu in the tissue is predominantly pro-inflammatory or anti-inflammatory. COX2, cyclooxygenase type 2; FLS, fibroblast-like synoviocyte; IL, interleukin; Mac, macrophage; MCP-1, monocyte chemoattractant protein 1; MHCII, major histocompatability complex class II; MMP, matrix metalloprotease; PDGF, platelet derived growth factor; RANTES, regulated on activation, normal T cell expressed and secreted; TGFβ, transforming growth factor β; Treg, T regulatory cell; VEGF, vascular endothelial growth factor. * Asterisk within the T cell denotes multiple subtypes: Th1, Th17, or Treg.

Cytokines serve as the mediators of cellular differentiation, inflammation, immune pathology, and regulation of immune response. A balance between pro-inflammatory and anti-inflammatory cytokines is essential for the development of a well-regulated effector immune response. The overproduction of pro-inflammatory cytokines and/or the deficiency of anti-inflammatory cytokines may lead to immune pathology [1,2,3,4,5]. The classic, well-known pro-inflammatory cytokines include tumor necrosis factor α (TNFα), interleukin-1β (IL-1β), IL-6, interferon-γ (IFN-γ) and IL-17 among others. TNFα, IL-1β and IL-6 are mainly produced by cells of myeloid origin such as macrophages and dendritic cells, whereas, IFN-γ and IL-17 are the defining cytokines for T helper 1 (Th1) and Th17 cells, respectively. Collectively, these pro-inflammatory cytokines, directly or indirectly, are involved in the differentiation and activation of pathogenic (e.g., Th17) cells, the migration of pathogenic cells into the target organ (the joints), the process of neovascularization (angiogenesis), the development and activation of osteoclasts, and the process of bone damage during the course of autoimmune arthritis [1,5]. On the other hand, the classic anti-inflammatory cytokines include IL-4 and IL-10, which display immunosuppressive activities. IL-23 and IL-27 represent additional cytokines of interest in rheumatoid arthritis (RA). IL-23 exerts pro-inflammatory activity with its ability to expand Th17 cells [6,7], whereas IL-27 acts as an anti-inflammatory cytokine in part via the inhibition of Th17 response [8,9]. The roles of most of the above-mentioned cytokines in the pathogenesis of RA are reviewed elsewhere by others and us [1,2,3,4], and are depicted in Figure 1. The salient features of three of the key pro-inflammatory cytokines (TNFα, IL-6, and IL-17) involved in arthritis are summarized below.

TNFα is produced primarily by macrophages but also can be produced by other cells, such as T cells, natural killer (NK) cells, mast cells and endothelial cells [10,11]. TNFα is released in large quantities when macrophages are exposed to lipopolysaccharide (LPS) or IL-1β [12]. TNFα is a member of the TNF superfamily [13]. The binding of TNFα to its receptor, the TNF receptor (TNFR) [14], can drive the cell to undergo apoptosis or activate the NF-κB/mitogen-activated protein kinase (MAPK) pathway. The latter pathway generates multiple mediators that participate in modulating apoptosis, cell survival, chemotactic migration of immune cells to the site of inflammation, and other inflammatory processes [11,14,15]. Neutralization of TNFα using either anti-TNFα antibodies or the soluble decoy receptor for TNFα has been instrumental in reducing the severity of arthritis, and a variety of biologics (e.g., infliximab, etanercept,* etc.*) based on this concept are currently being used in the clinic for the treatment of RA (Table 1). IL-17 is a critical pro-inflammatory cytokine in arthritis pathogenesis. Six members of the IL-17 family are named IL-17A-F. Among these, IL-17A has dominant role in the pathogenesis of RA. Th17 cells are the major source of IL-17A. The receptor complex consisting of IL-17RA and IL-17RC recognizes IL-17A and mediates downstream signaling events. These events in turn mediate upregulation of pro-inflammatory cytokines, chemokines, growth factors, and some enzymes of metabolic pathways, and alter oxidative status of the target organ. The impact of these events is manifest in the differentiation of naïve T cells into pathogenic T cells, the migration of pathogenic cells into the synovium, the increased survival of synoviocytes, angiogenesis, osteoclast differentiation and MMP secretion leading to bone and cartilage damage [16,17]. The expression of IL-17A is increased in inflammatory arthritis [16], and neutralization of IL-17A has been shown to reduce the severity of arthritis in the CIA model of RA [18]. Antibodies against IL-17 are currently being investigated in clinical trials for the treatment of RA (Table 1).

**Table 1:** Examples of antibodies tested for the treatment of arthritis and other inflammatory/autoimmune diseases.

Target	Agent	Type	Mechanism of Action	Ref.
IL-1	Anakinra ^a^	Recombinant IL-1Rα	Prevents binding of IL-1β to IL-1Rα	[19]
	Anti-IL-1β ^c^	Human IgG1 monoclonal antibody (mAb)	Binds tightly to IL-1β and neutralizes it	[20]
	Canakinumab ^b^	Human IgG1 mAb	Neutralizes the activity of IL-1β	[21]
	Gevokizumab ^b^	Humanized IgG2 mAb	Neutralizes the activity of IL-1β	[22]
	LY2189102 ^b^	Humanized IgG4 mAb	Neutralizes the activity of IL-1β	[23]
	Rilonacept ^b^	Ligand-binding domain of IL-1RI and human IgG1 fusion protein	Attaches to and neutralizes circulating IL-1β before it can bind its receptor	[24]
IL-6	MAB406 ^c^	mAb	Blocks IL-6 signaling	[ 25,26]
	MRA ^b^	Humanized mAb	Inhibits IL-6 signaling	[27]
	Tocilizumab ^a^	Humanized mAb	Binds to IL-6Rα chain and blocks IL-6 signaling	[28,29]
TNFα	Infliximab ^a^	Recombinant IgG1 mAb	Binds to TNFα and prevents it from binding to its receptor	[30,31]
	Adalimumab ^a^	Recombinant IgG1 mAb	Binds to TNFα and prevents it from activating TNF receptors	[32]
	Etanercept ^a^	Extracellular domain of TNF receptor II (p75) and the Fc portion of IgG1 fusion protein	Functions as a decoy receptor to TNFα	[33]
	Golimumab ^b^	Human mAb	Neutralizes TNFα bioactivity	[34]
IL-17	Secukinumab ^b^	Human IgG1k mAb	Selectively binds and neutralizes IL-17A	[35,36]
	Ixekizumab ^b^	Humanized IgG4 mAb	Neutralizes IL-17A	[37]
	Brodalumab ^b^	Human anti-IL17RA mAb	Inhibits the activity of IL-17	[38]
IL-12/IL-23	Ustekinumab ^b^	Human IgG_1κ_ mAb	Blocks the biologic activity of IL-12 and IL-23 through their common p40 subunit by inhibiting their receptors	[39]
	Guselkumab ^b^	Human mAb	An IL-23p19-targeted mAb	[40]

The current status of these biologics is given as ^a^ approved for treatment; ^b^ completed or in clinical testing; and ^c^ completed or in preclinical testing.

IL-6 has significant implications in the development of arthritis [41]. IL-6 is mainly produced by dendritic cells and macrophages. IL-6 triggers signaling by binding to IL-6 receptor (IL-6R), which is expressed on some leukocytes. A soluble form of IL-6R exists in serum. The complex of IL-6 and IL-6R, or the complex of IL-6 and soluble IL-6R binds to gp130, leading to the activation of signaling cascade. IL-6 contributes to RA pathogenesis via multiple effector responses including the differentiation of Th17 cells, osteoclast differentiation through expression of RANKL, and production of other pro-inflammatory mediators and tissue-degrading enzymes [41,42]. Neutralizaton of IL-6 represents a promising therapy for RA and biologics based on IL-6 are either currently being used for therapy or are in clinical testing (Table 1).

Table 1 shows a panel of therapeutic agents that target the key pro-inflammatory cytokines in RA. These agents can be categorized into three groups. The first group of agents are those that are currently being used in the management of RA. The safety profile and efficacy of these agents has been duly validated leading to their regulatory approval for RA therapy. The second group of agents has either completed clinical testing or is currently being investigated in clinical trials. Following this step, additional efficacy and safety requirements may have to be met prior to their regulatory approval for RA therapy. The third group contains agents that have been tested in animal models of RA only, but have not yet moved on to clinical testing in RA patients. It is hoped that some of them will soon be ready for clinical testing. Taken together, there is a pipeline of potential therapeutic agents that will expand the pool of reliable drugs for RA patients in the near future.

**Figure 2:**
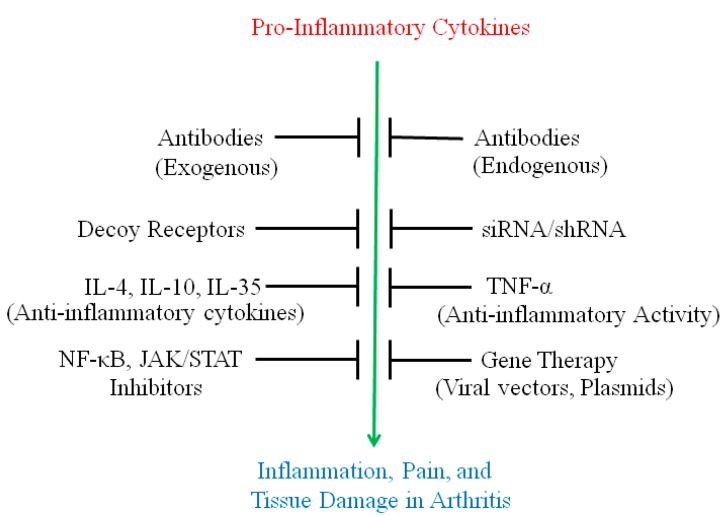
Diverse strategies employed to control the activity of pro-inflammatory cytokines and tissue damage. Pro-inflammatory cytokines, when produced in excess, can cause significant damage to tissues in various autoimmune diseases. Multiple approaches have been developed to prevent and ameliorate the harmful side effects of the pro-inflammatory cytokines. Anti-cytokine antibodies can inhibit the binding of the cytokines to their receptors. Decoy receptors can similarly bind the cytokines and prevent them from binding to the corresponding natural receptors on the cell surface. Gene therapy can be used to suppress the production of specific pro-inflammatory cytokines, whereas siRNAs can silence particular mRNA that encode the cytokine and thereby, prevent its production. Small molecule inhibitors can target certain pathways involved in the production of pro-inflammatory cytokines as well as inhibit their signaling abilities. Finally, anti-inflammatory cytokines help downregulate the pathogenic immune responses and subsequently inhibit further tissue damage. Paradoxically, TNFα can display anti-inflammatory properties under certain conditions.

In this article, we describe a variety of approaches used for modulation of cytokine responses to control arthritis (Figure 2, Table 1 and Table 2) and the properties of relatively newer cytokines (IL-32, IL-34, and IL-35), which have shown association with RA pathology and are being tested for their use in arthritis therapy in experimental models of RA.

**Table 2:** Cytokine modulation by gene therapy for arthritis therapy.

Target Cytokine	Vector	Mode of Gene Transfer	Model	Ref.
IL-1β	rAAV-IL-1Ra	*In vivo*	Mouse	[43]
	*IL-1Ra* Gene	*Ex vivo*	Human	[44]
	MFG-IRAP	*Ex vivo* modified Fibroblast	Human	[45]
TNF-α	sTNFR plasmid electrotransfer	*In vivo*	Mouse	[46,47]
	AAV-TNF-α	Locally into joints	Human	[48]
IFN-β	Adenovirus vector	Intra-articular	Rat	[49]
IL-4	Adenoviral vector	*In vivo*	Mouse	[50]
IL-10	Lentivirus using inflammation promoter switches Saa3 and Mmp13	Intra-articular	Mouse	[51]
IL-18	Adenoviral vector	*In vivo*	Mouse	[52]

## 2. Use of Antibodies and Decoy Receptors against Pro-Inflammatory Cytokines/Cytokine Receptors for the Treatment of Arthritis

A comprehensive understanding of the role of cytokines in the pathogenesis of RA has led to the development of new therapeutic approaches aimed at specifically neutralizing the cytokine or inhibiting cytokine signaling pathways. Unlike generalized immunosuppression, selective targeting of a particular pathogenic cytokine offers a distinct advantage for the treatment of RA and some other autoimmune diseases. The main target cytokines of interest for the treatment of RA are: TNFα, IL-1β, IL-6, IL-17, and IL-23 [1,5,53,54]. Inhibition of the activity of some of these cytokines has yielded promising results in studies on animal models of RA and in clinical trials in patients with RA [5,55]. Various biologics that target cytokines or cytokine receptors are comprised of specifically engineered (e.g., humanized) monoclonal antibodies, soluble recombinant cytokine receptors, and fusion-proteins containing the cytokine binding domain and the Fc portion of human IgG1. The nature and mechanisms of actions of different biologics that have been used for the treatment of RA and some other autoimmune diseases are summarized in Table 1.

Although efficacious, the above-mentioned biologics have some limitations [55,56,57,58]. These include the unresponsiveness of a proportion of patients to the therapeutic agent; the short-lived effect necessitating frequent injections of the biologic; gradual loss of sensitivity to the therapeutic agent with time; the likelihood of disease exacerbation (flares) if therapy is stopped for a long time; the high cost; and the increased risk of infections because of systemic immunosuppression. Given the limitations of anti-TNFα and anti-IL-1β therapy, continued efforts are directed towards targeting other cytokines (e.g., IL-6 and IL-17). Contrary to the expectation that perhaps combination therapy with different biologics might offer an advantage over a single agent, such an approach was found to impair the host defense without offering any additional advantage to RA patients [59].

Paradoxically, TNFα, which represents a prototypic pro-inflammatory mediator, can exhibit anti-inflammatory effects under certain conditions. For example, TNFα has been shown to suppress inflammation in rat AA, an experimental model of RA [60,61]. When Lewis (LEW) rats were immunized with heat-killed *Mycobacterium tuberculosis* (Mtb) for disease induction and then subsequently injected with TNFα i.p., these rats, when compared to controls, displayed a significant decrease in the severity of AA. Also, the amount of IFN-γ secreted in response to the pathogenic determinant of the disease-related antigen, mycobacterial heat-shock protein 65 (Bhsp65), was also lower in the TNFα-treated rats when compared to the controls [60,61]. Similarly, the* in vivo* regulatory role of TNFR p55 in Yersinia-induced arthritis in mice has been reported [62]. In another study, the exposure of eye-derived antigen-presenting cell (APC) to transforming growth factor β (TGFβ) resulted in increased expression of TNFα and TNFR2. This increase in expression was necessary in order to induce tolerance [63]. Furthermore, murine macrophages treated with TNFα produced less IL-23 and IL-12p70 after stimulation with IFN-γ and lipopolysaccharide (LPS), thus reflecting the anti-inflammatory effect of TNFα [64]. On the basis of the above finding, it is conceivable that some patients treated with neutralizing antibodies against TNFα (described above) might unexpectedly show aggravation of arthritis. This may occur if TNFα neutralization is performed under conditions that otherwise facilitate anti-inflammatory activity of endogenous TNFα. In view of the dual role of TNFα, above studies highlight that there is still much more to learn about the diverse functional attributes of these established cytokines in the pathogenesis of arthritis and other inflammatory disorders.

A new therapeutic approach based on cytokine inhibition is represented by active immunization as an alternative to passive immunization involving exogenous anti-cytokine antibodies [65,66]. Active immunization using synthetic peptides (epitope regions) of cytokines [67], recombinant cytokine containing T helper epitopes [68], or naked DNA [69] encoding the molecule have been shown to induce anti-cytokine antibodies, which can neutralize the cytokines produced* in vivo*. Active immunization strategies targeting the cytokines IL-1β [67], TNFα [68], and IL-23p19 [70], and the chemokine IFN-gamma-inducible protein 10 (IP-10) [69] have yielded promising results in animal models RA.

## 3. The Use of Small Molecule Inhibitors of Cytokine Production for Controlling Inflammation

A variety of natural inhibitors of cytokine signaling exist in the cells. These include members of the suppressor of cytokine signaling (SOCS), SH2-containing phosphatases (SHP), and the protein inhibitors of activated STATs (PIAS) families [71]. However, these inhibitors fail to effectively control chronic inflammation in patients with inflammatory diseases. Over the years, continuous efforts have been made to develop approaches that can prevent the production of a pro-inflammatory cytokine and thereby preempt subsequent cytokine-driven reactions and the resulting inflammation. Accordingly, many different molecules are known to inhibit NF-κB signaling by reducing the activation of the IκB kinase (IKK) complex by binding to the ATP-binding site of IKKβ [72]. Another effective target for inhibition is the Januk kinase (JAK) pathway. The molecule CP-690550, which is now known by the name Tofacitinib, is a JAK3 inhibitor. Rats/mice treated with this drug showed a marked reduction in the incidence and severity of arthritis in the adjuvant arthritis (AA) and collagen-induced arthritis (CIA) models of RA [73]. Multiple phase III trials have been conducted using Tofacitinib in RA patients [74]. These trials demonstrated the safety and efficacy of Tofacitinib. However, there were some side effects associated with its use. For example, studies in the mouse models have revealed the likely risk of activating latent tuberculosis [75].

Likewise, Celastrol, a natural bioactive compound derived from *Celastrus aculeatus* Merr, inhibits NF-κB activation and STAT3 signaling leading to the inhibition of IL-17, IL-6, IL-1β, TNFα, and chemokines, which results in the suppression of AA in rats [76,77,78]. TAK-242 (or Resatorvid) is a small molecule that inhibits Toll-like receptor 4 (TLR4) signaling by binding selectively to TLR4 and inhibiting its ability to associate with its adaptor molecules [79]. This inhibition prevents cells from becoming activated and producing pro-inflammatory cytokines. There are many small molecule inhibitors of cytokine production being tested besides those mentioned above [80].

## 4. Gene Therapy for Modulating Cytokine Response to Control Arthritis

Gene therapy permits sustained expression of gene products at precise anatomical locations [81,82,83,84], and such approaches aimed at correcting the cytokine balance have been tested in experimental models of RA and patients with RA [81,85,86]. In these approaches, the genes encoding specific products with anti-arthritic activity are delivered into intra- or extra-articular sites using viral or non-viral vectors. The targeting of various cytokines via gene therapy is summarized in Table 2 followed by a description of the silencing of specific genes for the purpose of modulating cytokine responses:

### 4.1. IL-1β

Various approaches have been developed to neutralize the effect of IL-1β by interleukin-1 receptor antagonist (IL-1Ra). Injection of recombinant adeno-associated virus vector encoding IL-1Ra (rAAV-IL-1Ra) complementary DNA [43] into the knee joint of rats was effective in producing optimal level of IL-1Ra locally and in suppressing arthritis in LPS-induced arthritis model. The IL-1Ra-encoding gene was among the first ones to be tested for potential use in a gene therapy clinical trial. In one study, the *IL-1Ra* gene was delivered locally into the metacarpophalangeal joints of a postmenopausal woman to test gene expression and production of IL-1Ra* ex vivo* [44]. In another study, the synovial fibroblasts collected from two RA patients were first transduced with a retrovirus, MFG-IRAP, carrying the IL-1Ra transgene and then were injected back into the inflamed metacarpophalangeal joints. Both patients responded to that treatment with reduced pain and swelling, and one of the patients showed reduced matrix metalloproteinase-3 (MMP-3) and IL-1β expression in synovial tissue tested* ex vivo* [45].

### 4.2. TNFα

Plasmids encoding soluble TNFα receptor (sTNFR) were transduced by electrotransfer and injected into mice with CIA. This treatment resulted in a decrease in both clinical and histological signs of the disease [46]. In another study in CIA, a similar treatment reduced clinical arthritis as well as IL-1β and IL-12 in the paws [47]. In a study in RA patients, the TNFα gene delivered using adeno-associated vector was found to be safe and well-tolerated [48].

### 4.3. IL-18

IL-18 is a pro-inflammatory cytokine that plays a role in the pathogenesis of RA [87,88] and it offers another cytokine target for RA gene therapy. Injection of an adenoviral vector containing the murine IL-18-binding protein (IL-18BP) gene into mice with CIA resulted in significant reduction in inflammation and destruction of bone and cartilage [52].

### 4.4. IFN-β

IFN-β possesses significant immunomodulatory properties and this cytokine has been considered for arthritis therapy [49,89]. Systemic administration of IFN-β was effective in suppressing arthritis in mice and monkeys, but did not have much effect in RA patients [89]. Therefore, approaches using local IFN-β treatment have been considered. In this regard, local IFN-β production by adenovirus-mediated gene transfer into the ankle joints of rats with AA resulted in the inhibition of arthritis progression and protection against bone damage [49].

### 4.5. IL-4 and IL-10

Gene therapy approaches using anti-inflammatory cytokines have also been attempted for arthritis control. Injection of an adenoviral vector encoding the gene for human IL-4 into mice resulted in decreased production of the inflammatory cytokines IL-1β and TNFα [50]. Injection of lentiviral vectors expressing the IL-10 gene under inflammation-dependent promoters such as Saa3 and Mmp13 intra-articularly into knee joints of mice resulted in reduced synovitis and cartilage proteoglycan depletion in experimental arthritis [51].

### 4.6. Small Interfering RNA (SiRNA)/Short Hairpin RNA (ShRNA)-Induced Cytokine Modulation for Arthritis Therapy

RNA interference, particularly employing siRNA, is a widely used approach to achieve the silencing of the gene of interest. TNFα genes delivered via nanoparticles consisting of polymerized siRNA targeting TNFα complexed with thiolated glycol chitosan polymer significantly inhibited inflammation and bone erosion in mice with CIA [90]. In another study based on a special type of delivery vehicle called wrapsome (WS), siRNA-encapsulating liposomes were systemically administered into mice with CIA. It resulted in significant reduction in the severity of arthritis as well as TNFα mRNA level in the joints [91]. The siRNA thus delivered was targeted primarily to CD11b^+^ myeloid cells. Similarly, poly(lactic-*co*-glycolytic) acid (PLGA) nanoparticles coated with arginine-glycine-aspartate (RGD) peptide and encapsulating STAT1-targeting siRNAs were effective in suppressing arthritis via selective inhibition of macrophage and dendritic cell activation [92]. Examination of the paws revealed reduced mRNA for STAT1 but increased mRNA for IL-10, an immunomodulatory cytokine for arthritis. In another study, the T-bet shRNA recombinant plasmid (*p*-T-shRNA), which was aimed at inhibiting T-bet, the transcription factor for Th1 cells, when delivered locally was effective in downregulating IFN-γ and IL-17 [93]. Despite the success of experimental approaches, siRNA therapy has not advanced into the clinic as rapidly as was anticipated earlier, in part owing to the sequence- and target-independent suppression and off-target effects [94,95].

Additional approaches for gene therapy of arthritis have been explored, including antigen-induced tolerance [96,97]. Application of gene therapy to modulation of cytokines offers a new approach for the management of RA and other types of arthritis. Despite several notable advancements discussed above, the goal of using gene therapy for effective symptomatic relief in RA without the side effects is still elusive. A summary of six clinical trials initiated in RA is described elsewhere [82]; however, none of these advanced beyond phase I. Most concerns in gene therapy have focused on the adverse effects, both short-term and long-term, caused by the viral vectors used [82,83,84]. It has shown that viral vectors may possess the risk of inducing an immune response, of vector spreading to other tissues, and of causing insertional mutagenesis, oncogenic transformations and lymphoproliferative disorders [82,83,84,98,99]. In fact, gene therapy was subjected to heavy scrutiny since it became known that the viral vectors used in a clinical trial subsequently caused leukemia [34]. However, many of these concerns have been overcome by several advancements in the type of viral vectors being used in gene therapy and by the availability of refined non-viral vectors [82,83,84]. In addition to the above-mentioned risks, there are some ethical considerations for gene therapy. In the case of somatic cell gene therapy, the ethical concerns relate to the relative benefit/risk ratio, criteria for selection of patients for experimental therapy, ensuring voluntary participation, and maintaining privacy and confidentiality of the procedures [100]. In the case of germ-line gene therapy, there is an additional concern that this approach might become a tool for enhancement of specific genetic traits in otherwise healthy individuals [101]. Nevertheless, although gene therapy may currently pose some risks, with new advancements and ethical regulations, it can be harnessed for treatment of arthritis and other chronic, debilitating diseases.

## 5. The Relatively Newer Cytokines (IL-32, IL-34 and IL-35) and Their Roles in Inflammation and Autoimmune Arthritis

### 5.1. IL-32

The IL-32 gene has been identified in most mammals except rodents [102]. IL-32 is produced by synovium-infiltrating lymphocytes and synovial fibroblasts of arthritic joints. In RA, the levels of IL-32 correlated with the severity of the disease as well as with the expression of other pro-inflammatory cytokines, including TNFα and IL-1β [103,104,105]. IL-32 plays a critical role in the differentiation and activation of macrophages [106]. Furthermore, IL-32 cooperates with TNFα and IL-17 in facilitating increased production of other pro-inflammatory cytokines and chemokines leading to chronic inflammation and osteoclastic bone erosion in RA [107]. IL-32 induces the production of IL-17 in CD4^+^ T cells, and reciprocally IL-17 affects the expression of IL-32 in fibroblasts-like synoviocytes (FLSs) of RA patients [108]. The overexpression of human IL-32β and the adoptive transfer of CD4^+^ T cells expressing this cytokine into mice led to aggravation of CIA [104]. Furthermore, intra-articular injection of IL-32 into naïve mice or into mice with CIA caused joint swelling, migration of inflammatory cells into the joints, and cartilage damage [103,109]. These effects of IL-32 were significantly reduced or abrogated in mice deficient in TNFα, indicating that some of the actions of IL-32 are TNFα-dependent [103].

### 5.2. IL-34

IL-34 shows a functional association with macrophage colony stimulating factor (M-CSF) [110,111], and it has been implicated in macrophage differentiation and osteoclastogenesis [112,113,114]. In addition, injection of IL-34 into mice increases the number of osteoclast precursors and enhances bone resorption [112]. Other pro-inflammatory cytokines such as TNFα and IL-1β can stimulate IL-34 expression through the NF-κB and JNK pathway [115], whereas IL-34 can increase the production of IL-17 [116]. IL-34 is expressed in the synovium and fibroblast like synoviocytes (FLS) of RA patients. Increased levels of IL-34 have been reported in serum and synovial fluid of RA patients, and the levels of IL-34 correlate well with that of IL-6, receptor activator of nuclear factor kappa-B ligand (RANKL), rheumatoid factor (RF) and anti-cyclic citrullinated peptide antibody (ACPA) [117].

### 5.3. IL-35

IL-35 is a member of the IL-12 cytokine family [118,119]. IL-35 is an anti-inflammatory and immunosuppressive cytokine produced by natural CD4^+^Foxp3^+^ regulatory T cells (nTreg). Interestingly, IL-35 can induce the differentiation of CD4^+^ effector T cells into a distinct subset of regulatory T cells, which in turn express IL-35 but do not express Foxp3, TGF-β and IL-10 (iTreg35 cells) [118]. Furthermore, such iTreg35 cells can suppress experimental autoimmune encephalomyelitis (EAE) and inflammatory bowel disease (IBD) as tested in animal models [118]. This suppression by iTreg35 was mediated via IL-35. Other investigators have also reported that IL-35, either injected as exogenous IL-35 or expressed locally* in vivo*, can prevent experimental autoimmunity such as CIA, type 1 diabetes (T1D), and colitis [120,121,122,123]. The protective effect of IL-35 against CIA involved the expansion of CD4^+^CD25^+^Foxp3^+^ Treg coupled with a reduction of IL-17 response but an increase in IFN-γ production [120]. In another study, IL-35-induced suppression of CIA was associated with stimulation of CD39^+^ CD4^+^ CD25^−^ regulatory T cells and an increase in IL-10 production, combined with a reduction in IL-17, IFN-γ, and anti-CII antibodies [121]. However, a recent study highlighted the contradictory disease-aggravating effect of IL-35. In that study, IL-35 gene transfer resulted in aggravation of arthritis (CIA) and increased the ratio of Th17/Treg cells in the spleen of arthritic mice [124]. Studies in other models of autoimmunity revealed that local expression of IL-35 in pancreatic β-islets can suppress T1D in non-obese diabetic (NOD) mice [122], and that IL-35 treatment can suppress colitis [123]. Thus, additional studies are required to further clarify the pro-* vs.* anti-inflammatory activity of IL-35.

As discussed above, some of the newer cytokines such as IL-32, IL-34 and IL-35 are being investigated for their role in the pathogenesis and treatment of arthritis. The predominantly pro-inflammatory members of this group can be targeted for their downmodulation, whereas the predominantly anti-inflammatory members of the group can be boosted for their therapeutic effects in arthritis. Various approaches, including antibody neutralization, gene therapy, transcriptional silencing,* etc.*, which already have been tested with established cytokines, can be extended to these newer cytokines (Figure 2). It is hoped that continued advancements in ongoing approaches coupled with novel cytokine targets will have a significant impact on the management of arthritis in the very near future.

## 6. Conclusions

Pro-inflammatory cytokines, which drive the initiation and progression of autoimmune arthritis, are the prime therapeutic targets for this debilitating disease. The biologics based on neutralization of a few of the pro-inflammatory cytokines are already in the clinic and additional new ones are in clinical/preclinical trials. Gene therapy targeting these cytokines is also gaining momentum despite overall general decline in interest in this approach over the past decade or so. Furthermore, newer cytokines are on the horizon, some pro-inflammatory and others anti-inflammatory. It is hoped that one or more of these cytokine might offer a viable therapeutic target for arthritis therapy. Overall, most of the effort in cytokine-based therapy is focused on dampening pro-inflammatory cytokines. However, there are some promising leads for accentuation of the anti-inflammatory/immunoregulatory cytokines to control autoimmune arthritis. In conclusion, there is high optimism for the development of new arthritis therapies based on advances in basic science aspects of this disease in the coming years.
